# P-465. Evaluating Urinary i-FABP as a Predictor of Clinical Outcomes in Preterm Infants ≤ 33 weeks with Complicated Intraabdominal Infection

**DOI:** 10.1093/ofid/ofaf695.680

**Published:** 2026-01-11

**Authors:** Taylor Kohlmann, Angel A Marks, Iyanuoluwa Ayodele, N David Yanez, Daniel Benjamin, Michael Cohen-Wolkowiez, Michael J Smith, P Brian Smith, Angelique E Boutzoukas

**Affiliations:** Duke University School of Medicine, Durham, North Carolina; Duke Clinical Research Institute, Durham, North Carolina; Duke Clinical Research Institute, Durham, North Carolina; duke university, Durham, North Carolina; Duke University School of Medicine, Durham, North Carolina; Duke University, Durham, North Carolina; Duke University, Durham, North Carolina; Duke University, Durham, North Carolina; Duke University/Duke Clinical Research Institute, Raleigh, NC

## Abstract

**Background:**

Complicated intraabdominal infections (cIAIs) cause significant morbidity and mortality in premature infants. Intestinal fatty acid-binding protein (i-FABP), a noninvasive biomarker of enterocyte death, has been shown to predict the occurrence and severity of necrotizing enterocolitis (NEC). Whether i-FABP predicts outcomes of preterm infants with cIAI is unknown.

**Table 1.**
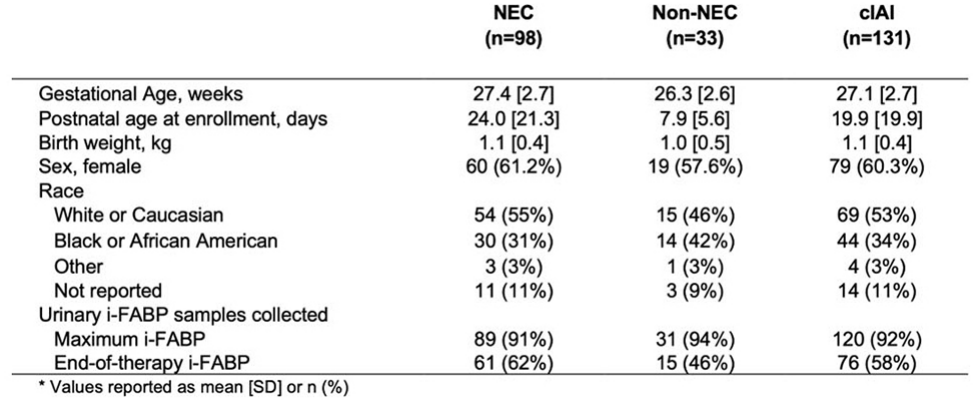
Characteristics of preterm infants with cIAI and at least one i-FABP measurement (N=131)

**Figure 1.**
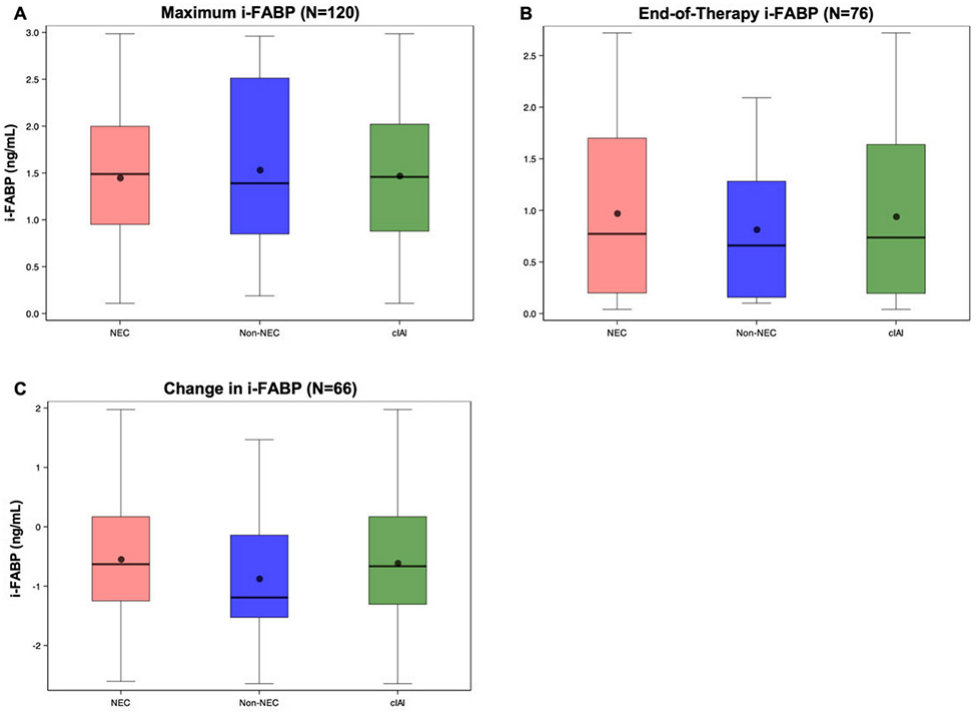
Maximum, End-of-Therapy, and Change in i-FABP in Preterm Infants with cIAI A. Distribution of maximum i-FABP (ng/mL) by cIAI diagnosis. From left to right: NEC vs. non-NEC vs. all infants with cIAI, mean [95% CI]: 1.45 [1.29, 1.61], 1.53 [1.21, 1.86], and 1.47 [1.33, 1.61], respectively. B. Distribution of end-of-therapy i-FABP by cIAI diagnosis. From left to right: NEC vs. non-NEC vs. all infants with cIAI, 0.97 [0.76, 1.19], 0.82 [0.46, 1.18], and 0.94 [0.76, 1.13], respectively. C. Distribution of change in i-FABP by cIAI diagnosis. From left to right: NEC vs. non-NEC vs. all infants with cIAI, -0.54 [-0.83, -0.26], -0.87 [-1.67, -0.07], and -0.61 [-0.88, -0.34], respectively.

**Methods:**

We included infants ≤ 33 weeks gestation, < 121 days postnatal age, and with ≥ 1 urinary i-FABP measurement from the Antibiotic Safety and Effectiveness in Premature Infants trial, a prospective study of infants with cIAI (NCT01994993, BPCA-sponsored). We quantified i-FABP in 3 ways: maximum (highest value within 5 days of antibiotic initiation), end-of-therapy (EOT, last value on or after day 7), and change (EOT minus maximum) in all infants with cIAI and in NEC vs. non-NEC subgroups. The primary outcome was a composite Clinical Cure Score (CCS) ≤ 4 at 30 days, indicating severe illness. Secondary outcomes were 30-day mortality and short bowel syndrome, intestinal perforation, or GI surgery within 90 days of antibiotic completion. We used logistic regression to estimate changes in odds of each outcome per 1 ng/mL difference in i-FABP.

**Figure 2.**
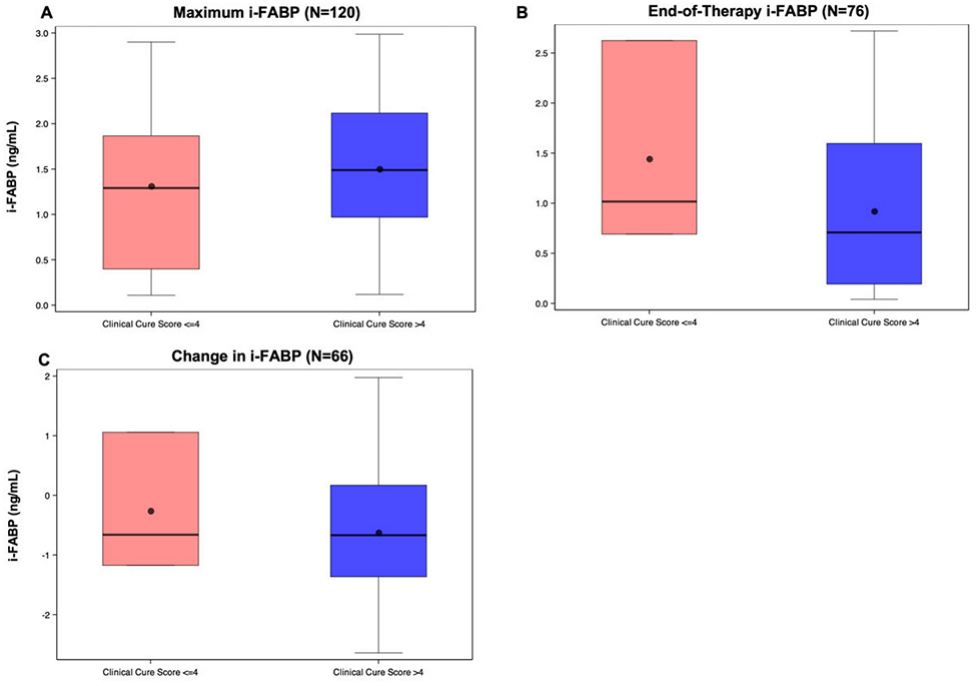
Maximum, End-of-Therapy, and Change in i-FABP by Clinical Cure Score at 30-days after treatment for cIAI

Clinical Cure Score: 1 point awarded for each of the following elements: FiO2 ≤ baseline FiO2, urine output ≥ 1 mL/kg/hour for 24 hours, no inotropic support, no mechanical ventilation, no seizure in 24 hours, and serum pH ≥ 7.25 or not measured. Scores ≤ 4 represent severe illness, and scores > 4 represent favorable clinical status. A. Distribution of maximum i-FABP values (ng/mL) by Clinical Cure Score (left to right: ≤ 4 vs. > 4; mean [95% CI]: 1.31 [0.90, 1.72], 1.50 [1.35, 1.66], and 1.47 [1.33, 1.61], respectively). B. Distribution of end-of-therapy i-FABP values by Clinical Cure Score (left to right: ≤ 4 vs. > 4; 1.44 [-1.13, 4.02], 0.92 [0.74, 1.11], and 0.94 [0.76, 1.13], respectively). C. Distribution of change in i-FABP values by Clinical Cure Score (left to right: ≤ 4 vs. > 4 vs.; -0.26 [-3.16, 2.65], -0.62 [-0.90, -0.35], and -0.61 [-0.88, -0.34], respectively.

**Table 2.**
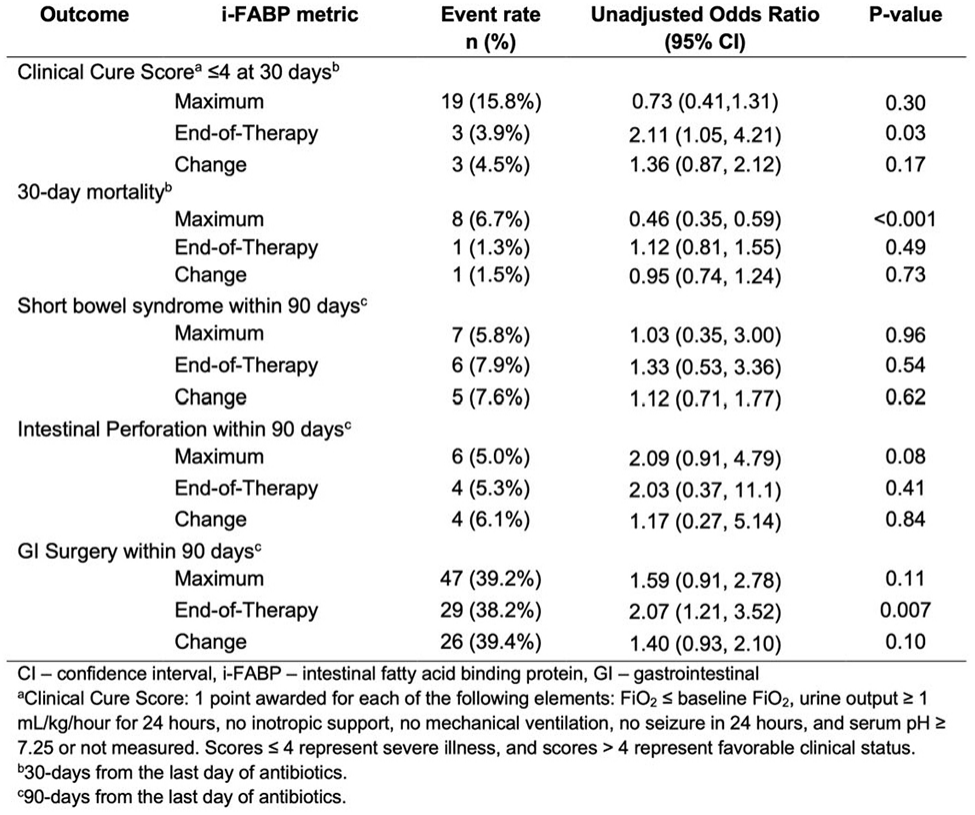
Unadjusted Event Rates and Odds Ratios for association of i-FABP and Clinical Outcomes in cIAI

**Results:**

131 eligible infants had 120 maximum, 76 EOT, and 66 change in i-FABP values (Table 1). i-FABP distributions were similar over time in NEC and non-NEC subgroups (Figure 1). In cIAI, while maximum and change in i-FABP did not vary substantially by illness severity (CCS), EOT values were higher in those with CCS ≤ 4 (Figure 2). Maximum and change in i-FABP were not associated with severe illness (CCS ≤ 4) at 30 days, while higher EOT values were associated with higher odds of CCS ≤ 4 (OR 2.11, 95% CI [1.05, 4.21], p = 0.03, Table 2). Maximum i-FABP was associated with lower 30-day mortality in cIAI (OR 0.46, 95% CI [0.35, 0.59], p < 0.001), while EOT and change were not. Higher EOT i-FABP was associated with higher risk of GI surgery by 90 days in cIAI (OR 2.07, 95% CI [1.21, 3.52], p = 0.007).

**Conclusion:**

Serial i-FABP measurements, especially at the end of therapy, may hold value in prognosticating outcomes of premature infants with cIAI, such as illness severity and need for GI surgery. Future work will define test performance of i-FABP as a predictor of cIAI outcomes.

**Disclosures:**

Daniel Benjamin, Jr., MD, PhD, MPH, AbbVie, Inc.: Advisor/Consultant|PPD, Inc.: Advisor/Consultant|Syneos Health: Advisor/Consultant Michael J. Smith, M.D., M.S.C.E, Pfizer: Grant/Research Support P. Brian Smith, MD MPH MHS, abbott nutrition: Expert Testimony|Biocryst: Advisor/Consultant|Syneos Health: Advisor/Consultant Angelique E. Boutzoukas, MD, MPH, Elion Therapeutics: Advisor/Consultant|Innoviva Speciality Therapeutics: DSMB Participant

